# Estimating Latent Tuberculosis Infection Using Interferon-γ Release Assay, Japan

**DOI:** 10.3201/eid2411.171948

**Published:** 2018-11

**Authors:** Tomoyasu Nishimura, Masaki Ota, Masaaki Mori, Naoki Hasegawa, Hiroshi Kawabe, Seiya Kato

**Affiliations:** Keio University, Tokyo, Japan (T. Nishimura, M. Mori, H. Kawabe);; Research Institute of Tuberculosis—Japan Anti-Tuberculosis Association, Tokyo (M. Ota, S. Kato);; Keio University School of Medicine, Tokyo (N. Hasegawa)

**Keywords:** Latent tuberculosis infection, foreign-born student, Japan, epidemiology, interferon-γ release assay, bacteria, tuberculosis and other mycobacteria, TB

## Abstract

We estimated the latent tuberculosis infection (LTBI) rate for foreign-born students at Keio University, Tokyo, Japan, using an interferon-γ release assay. The LTBI rate for students from countries with estimated tuberculosis incidence >100 cases/100,000 persons was high (10.0%). Universities should screen for LTBI in students from countries with high tuberculosis incidence.

The proportion of foreign-born tuberculosis (TB) patients among all TB patients in Japan is increasing, particularly for those 20–29 years of age (57.7% in 2016) (*1*). The Tokyo metropolitan government revealed a foreign-born student–related TB outbreak at a Japanese language school in 2016 (*2*). TB outbreaks involving foreign-born students create concerns that TB infection from such students, particularly those from countries with a high incidence of TB, might spread to the population of Japan. 

In Japan, university students, including foreign-born students, undergo TB screening with chest radiograph; however, a chest radiograph cannot detect LTBI; it detects only pulmonary TB. Because immigrants may develop TB after entry (*3*), screening with chest radiograph might be ineffective; therefore, screening for LTBI may be necessary to prevent TB outbreaks. However, only a few surveys of TB infection among foreign-born persons have been conducted in Japan (*4*). We conducted a survey of LTBI among foreign-born students by using an interferon-γ release assay (IGRA).

Keio University has 6 campuses in the greater Tokyo area comprising ≈33,000 students, of whom ≈1,600 are foreign-born from 74 countries. During September 2016–September 2017, we recruited foreign-born students >20 years of age studying at Keio University who had no history of mycobacterial diseases or HIV infection. After obtaining informed consent, we collected whole blood specimens for the T-SPOT.TB test (Oxford Immunotec Ltd., Abington, UK), an IGRA available in Japan. All participants were screened for pulmonary TB with chest radiograph. We interviewed participants using a structured questionnaire on identification and demographic information, the date of first arrival in Japan, and history of TB. We derived country-specific estimated TB incidence rates from the World Health Organization website (*5*). Statistical results were computed by using R software (The R Foundation, Vienna, Austria). This study was conducted in compliance with the Declaration of Helsinki and approved by the institutional ethics review committee for human research of the Keio University School of Medicine and Hospital (no. 20160080).

We enrolled 177 participants 20–42 years of age (median 23 years), of whom 98 (55.1%) were female (Table). Participants were from China (55 students), Indonesia (24 students), France (19 students), Germany (9 students), and Thailand (8 students); the remaining participants were from 28 different countries, including 50 from countries with estimated TB incidence rates >100 cases/100,000 persons. We excluded data for 1 participant with an indeterminate IGRA result. A total of 117 (66.1%) students participated in this study within 1 month after arriving in Japan.

**Table:** Characteristics and IGRA results of participants in a study of latent TB infection, Japan, 2016–2017*

Characteristic	IGRA positive, no. (%, 95% CI), n = 8	IGRA negative, no., n = 169	p value†
Sex			
F	5 (5.1, 1.7–11.5)	93	0.733
M	3 (3.8, 0.79–10.7)	76	
Age, y			
20–29	6 (3.9, 1.4–8.3)	148	0.278
>30	2 (8.7, 1.1–28.0)	21	
TB incidence rate in country of origin			
<100 cases/100,000 population	3 (2.4, 0.49–6.7)	124	0.042‡
>100 cases/100,000 population	5 (10.0, 3.3–21.8)	45	
Time living in Japan, y			
<1	5 (3.4, 1.1–7.9)	140	0.158
>1	3 (9.4, 2.0–25.0)	29	

*IGRA, interferon-γ release assay; TB, tuberculosis. †Differences between positive and negative groups were tested using Fisher exact test. ‡p<0.05.

Overall, 8 (4.5% [95% CI 2.0%–8.7%]) students tested positive on IGRA (2 each from China and Thailand and 1 each from Ghana, Indonesia, South Korea, and the Philippines). The rate of the positive IGRA result for students from countries with an estimated TB incidence rate of >100 cases/100,000 persons was 10.0% (95% CI 3.3%–21.9%) and relative risk was 4.2 (95% CI 1.1–17.1), whereas the rate for students from countries with an estimated TB incidence rate of <100 cases/100,000 persons was 2.4% (95% CI 0.49%–6.7%). Even IGRA positivity of students 20–29 years of age from countries with estimated TB incidence rates of >100 cases/100,000 persons was 9.4% (95% CI 2.0%–25.0%). Chest radiograph found no students with pulmonary TB. We recommended that all IGRA-positive students receive LTBI treatment and close follow-up to detect the development of TB as early as possible.

The overall rate of LTBI among foreign-born students at Keio University was 4.5%. This rate was significantly higher for these students than for Keio University students from Japan assessed during 2009–2013 (0.73% [95% CI 0.39%–1.2%]; T. Nishimura et al., unpub. data). Our findings are consistent with those of previous studies. Ogiwara et al. showed that 7.8% of study participants tested positive for LTBI using the QuantiFERON-TB Gold test on 384 foreign-born students, of whom 363 were from countries with high TB incidence rates (*4*).

Our study had a few strengths and limitations. The number of study participants was large enough for us to stratify the participants by estimated TB incidence rates for their countries of origin. One limitation was that the participation rate was small. Just ≈11% of foreign-born students at Keio University participated; therefore, the results obtained might not be representative of LTBI in all foreign-born students.

In conclusion, we found that estimated LTBI rates for foreign-born students in Japan from countries with high TB incidence rates were higher than those for students from countries with low TB incidence rates and for students from Japan. Based on our findings, we recommend that universities screen for LTBI using IGRAs in students from countries with high TB incidence rates (i.e., >100 cases/100,000 persons).
